# Immunomodulation by platelet-derived DKK1: potential for controlling disease and pathology in leishmaniasis and implications for other infectious diseases

**DOI:** 10.3389/fimmu.2026.1862987

**Published:** 2026-06-02

**Authors:** Olivia C. Ihedioha, Diane McMahon-Pratt, Alfred L. M. Bothwell

**Affiliations:** 1 Department of Pathology, Microbiology, and Immunology, University of Nebraska Medical Center, Omaha, NE, United States; 2 Department of Epidemiology of Infectious Diseases, Yale School of Public Health, New Haven, CT, United States; 3 Department of Immunobiology, Yale University, New Haven, CT, United States

**Keywords:** Dickkopf1, diseases, immune response, *Leishmania* major, α-granules

## Abstract

Parasitic infection caused by *Leishmania major* in BALB/c mice is a well-established example of a chronic inflammatory disease. Although chronic inflammation in parasitic infections stems from persistent interactions between parasites and host immune cells, the mechanisms by which parasitic infections induce and regulate chronic immune responses remain to be fully understood. Emerging evidence suggests that platelets contribute not only to hemostasis but also modulate immune response during infection and inflammation. This review examines the emerging role of platelet-derived Dickkopf-1 (DKK1), an inhibitor of Wnt signaling, in shaping immunity during cutaneous leishmaniasis and explores its broader implications in other infectious diseases. Experimental findings from BALB/c mouse models indicate that *Leishmania major* infection promotes platelet activation and DKK1 release, leading to enhanced leukocyte-platelet aggregation and recruitment of neutrophils, macrophages, and dendritic cells to inflammatory sites. Platelet-derived DKK1 appears to influence dendritic cell polarization, favoring cDC2 and DC-10-mediated T-cell differentiation associated with Th2 and regulatory immune responses which contribute to M2 macrophage polarization and intracellular parasite survival. In contrast, protective antileishmanial Th1-associated responses may be diminished in the presence of sustained DKK1 signaling. Thus, this review integrates current evidence indicating that platelet-derived DKK1 functions as an early regulator of both innate and adaptive immune responses during *Leishmania* infection. Beyond leishmaniasis, accumulating evidence suggests that platelet activation and DKK1 release may also participate in the immunopathology of fungal and viral infections. By integrating current evidence on platelet-mediated immune regulation, this review highlights platelet-derived DKK1 as a potential immunomodulatory target and emphasizes the need for further studies to clarify its translational relevance across infectious diseases.

## Graphical Abstract

**Figure:**
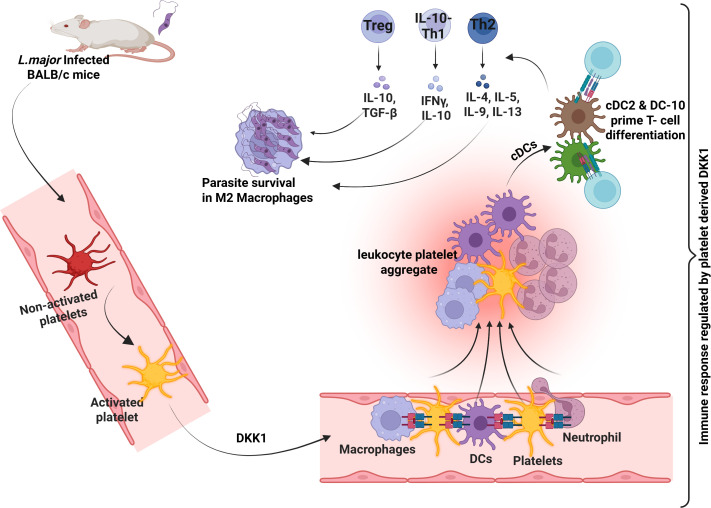

Schematic illustration of immunomodulation by platelet DKK1 leading to suppression of anti-leishmanial immune response and parasite survival in BALB/c infected mice. Created in BioRender. Ihedioha, O. (2026) https://BioRender.com/3u47csf

## Wnt signaling and immune regulation

The Wnt signaling pathway is established as a highly conserved signaling network that regulates multiple cellular processes (cell proliferation, differentiation, migration and survival), embryonic development, and tissue homeostasis (1–3). The pathway is generally categorized into the canonical and non-canonical branches according to the β-catenin’s involvement in transcriptional activation (4). The canonical Wnt pathway is defined by the stabilization and nuclear translocation of β-catenin, which subsequently interacts with T cell factor/lymphoid enhancer factor (TCF/LEF) transcription factors to activate target gene expression, primarily promoting cell proliferation. In contrast, the non-canonical Wnt pathway acts independently of the β-catenin–TCF/LEF signaling axis, regulating processes such as cell polarity and migration while interacting with canonical signaling through a complex and interconnected regulatory network (1, 5). At the cell surface, Wnt proteins bind to Frizzled receptors and LRP5/6 co-receptors triggering the canonical β-catenin-dependent pathway (3, 6).

Beyond its developmental role, Wnt signaling pathway proteins are increasingly recognized as a critical regulator of immune function and inflammatory responses. Previous immunological studies have highlighted that Wnt signaling pathways and Wnt ligands have a crucial and substantial role in regulating immune cell functions. This signaling system exhibits broad functional diversity across immune populations, including promoting the tolerogenic properties of dendritic cells, supporting natural killer cell development, driving T-cell maturation during thymopoiesis, facilitating B cell-mediated activation of T cells, and modulating macrophage activities involved in tissue repair, regeneration, and fibrotic processes (7).

Thus, the canonical Wnt/β-catenin signaling generally promotes immune tolerance and resolution of inflammation, whereas inhibition of this pathway can enhance inflammatory cytokine production and leukocyte recruitment (8). Wnt signaling also contributes to host-pathogen interactions by modulating antimicrobial responses, tissue remodeling, and immune evasion mechanisms exploited by pathogens (9–11). This complex network of interactions ensures precise controlled regulation in both physiological state and pathological conditions (12). Previous studies in both mice and humans has linked abnormal regulation of Wnt signaling pathways, as well as altered expression of Wnt inhibitors, with diseases such as cancer, asthma, and autoimmune diseases (13–15).Wnt ligands and antagonists, including DKK1, influence the activation, differentiation, and polarization of immune cells (7).

## Dickkopf1 family

The DKK protein family is an ancient, evolutionarily conserved gene family that consists of four main secreted proteins (DKK-1, -2, -3, -4) and the DKK-3-related protein soggy (Sgy-1 or DKKL1) (16, 17). Unlike the four primary DKK secreted proteins, which typically range from 255 to 350 amino acids in length, soggy (DKKL1) is shorter, consisting of a 242–amino acid protein due to its unique structural features (16, 17). These glycoproteins play crucial roles in various biological processes, especially in modulating the Wnt signaling pathway. The DKK protein family harbors an N-terminal signal peptide and two conserved cysteine-rich domains (CRD1 and CRD2). CRD1 is located toward the N-terminus and is unique to the human DKK family of proteins (18). Both CRD1 and CRD2 are required for receptor binding at the cell surface. The N-terminal cysteine-rich region of DKK proteins (DKK-N, formerly known as Cys1) is unique to the DKK family. The C-terminal cysteine-rich region (formerly known as Cys2) is a colipase fold containing 10 cysteine-related patterns (19). The colipase fold consists of short β-strands, which are linked by loops and stabilized by disulphide bonds to form finger-like structures preserved as surface interactions. The non-conservative junction spanning 50–55 amino acids separates DKK-N from the colipase fold. Cys2 binds to low-density lipoprotein-related protein 6 (LRP6) and Kremen2, thereby blocking the Wnt pathway (20).

## DKK1 structure

DKK1 (an important member of the DKK protein family) is a secretory glycoprotein that was first discovered in the embryonic cells of Xenopus (16). The DKK1 gene is located on chromosome 10q11 and is about 3.5 kb long. DKK1 protein consists of 266 amino acids, with a relative molecular weight of 29 kDa. The DKK1 receptor (LRP5/6) contains four tandem YWTD-type β-propeller and EGF-like domains, designated LRP6 (E1–E4) (21, 22). These extracellular LRP5/6 domains facilitate the binding of Wnt ligands as well as their inhibitors, including DKK1. For instance, Wnt1, Wnt2, Wnt7a, Wnt7b, Wnt9a, Wnt9b, Wnt10a, and Wnt10b bind to the E1 domain of LRP6, whereas Wnt3 and Wnt3a associate with the E3 domain (21, 23, 24). Structural studies of DKK1 and LRP6 revealed that LRP6 contains several Wnt-binding domains that can be occupied by DKK1, thereby blocking Wnt ligands from interacting with the receptor (21, 22, 24, 25). Specifically, the C-terminal domain of DKK1 binds to LRP6 (E3–E4), while its N-terminal domain interacts with LRP6 (E1–E2) (21). Although DKK1 is capable of binding to both regions of the receptor ectodomain (E1E2 and E3E4), its C-terminal interaction with the LRP6 E3–E4 domains is consistently recognized as the dominant high-affinity binding event responsible for inhibition of Wnt signaling. This dual interaction between DKK1 and LRP6 indicates that DKK1 can inhibit multiple Wnt proteins. Thus, DKK1 potentially can act to limit activation of the Wnt/β-catenin pathway. Because Wnt signaling is essential for tissue repair and immune homeostasis, sustained DKK1 production may exacerbate pathological inflammation and impair resolution.

## DKK1 expression

DKK1 expression is elevated during embryogenic development but remains relatively low in most adult tissues. It is secreted by various cell types, such as osteoblasts and mesenchymal stem cells, and contributes to bone formation in normal physiological processes (26–28). However, in disease-related scenarios, excessive DKK1 expression has been linked to various conditions, including cancer (29–34). Cancer-associated fibroblasts (CAFs) and other stromal cells within the tumor microenvironment are recognized as sources of DKK1 production (33).

Higher concentrations of DKK1 in the bloodstream are linked to chronic inflammatory conditions (31, 35–37). Previous studies demonstrated that inflammatory cytokines induce intestinal epithelial cells to produce DKK1 during colitis (38) and also in response to *Cryptosporidium parvum* infection (39). Inhibition of DKK1 function results in elevated Wnt signaling and the induction of cell proliferation, which promotes wound repair after colitis (38). Further, DKK1 has been found to be elevated in cases of pediatric infectious diseases (40). A recent study also highlighted DKK1 (induced by amyloid-β in the intact rodent brain) as a key molecule in the pathogenesis of Alzheimer’s disease; blocking the DKK1-LRP6 signaling pathway inhibits cognitive impairment (41).

## Platelets as early immune modulators in infection

Platelets are increasingly recognized as early immune modulators that actively participate in host defense during infection, beyond their classical role in hemostasis (42–44). Upon activation, they release a wide range of bioactive mediators from their granules that shape local inflammatory responses (45, 46). As one of the first circulating cells to encounter invading pathogens, platelets rapidly respond through pattern recognition receptors, including Toll-like receptors (TLRs), C-type lectin receptors, and complement receptors (47–49).

During inflammation, platelets interact closely with leukocytes, promoting leukocyte recruitment, neutrophil extracellular trap (NET) formation, monocyte activation, dendritic cell maturation, and modulation of macrophage polarization (47, 50). Platelet-leukocyte aggregates are now recognized as important amplifiers of innate immune signaling and inflammation responses (51–53). In addition, platelets contribute to the regulation of adaptive immunity by influencing antigen presentation and T-cell polarization through soluble mediators and direct cellular interactions (53). Platelets are increasingly recognized as key modulators of host-pathogen interactions across bacterial, viral, fungal, and parasitic infections (44, 47, 54). Their responses can be either beneficial or detrimental depending on the disease context. Although platelet activation can promote pathogen clearance and support tissue repair, uncontrolled or excessive platelet-mediated inflammation may drive immunopathology and impaired immune function. (53, 55).

## Platelet-derived DKK1 regulation of homeostatic and inflammatory conditions

While DKK1 is known to be secreted by osteoblasts and cancer cells, it is also secreted by some immune cells, with platelets being a notable example (54, 56, 57). The clinical significance of activated platelets as a DKK1 producer is substantiated by low circulating DKK1 levels in aspirin users (58). Beyond their classical role in hemostasis, activated platelets rapidly release DKK1 from α-granules in response to inflammatory stimuli, pathogen-associated molecules, and vascular injury (54, 56, 59). Platelet-derived DKK1 acts as a critical, secreted regulator of both tissue homeostasis and inflammation. Under homeostatic conditions, PRP (platelet rich plasma) is known to contain more than 20 growth factors that play roles in spinal infusion (60), fracture healing (61), wound healing (62), bone defect repair (63) and bone regeneration. In the context of bone regeneration, the effects of PRP are often described as controversial due to the evidence that PRP with a high concentration of growth factors leads to lesser osteogenic responses (64–66). This suggests that PRP’s reduced efficacy in bone regeneration may be due to a platelet-derived anti-osteogenic factor that inhibits the osteoblast response to other platelet-derived growth factors. PRP is rich in DKK1, and DKK1 is established as an anti-osteogenic factor known to inhibit bone regeneration after bone damage by blocking Wnt signaling (67). Experimentally, bone fracture healing is impaired by DKK1 (implying that the biologic role of DKK1 is to prevent excessive healing responses induced by the Wnt signaling pathway) (68, 69). Normal DKK1 expression is reported to have a positive impact, promoting a stable, non-progressive osteoarthritis in women (70). However, overexpression of DKK1 has been found to negatively affect the joint, leading to a bone-destructive pattern, loss of bone mineral density, and cartilage degradation (71). These findings suggest that platelet DKK1 may play a role in joint and bone function, and that maintaining a balanced level of platelet-derived DKK1 in the joint environment is crucial for optimal joint health. Given the multiple mechanisms by which platelet DKK1 influences bone regeneration and joint environment, it could be a useful diagnostic marker or a treatment for joint diseases in humans and animals.

## Platelet-derived DKK1 and viral infection

Recent study demonstrated that platelet-derived DKK1 contributes to the thrombo-inflammatory immune dysregulation observed during coronavirus infection, particularly in COVID-19 (72). It was demonstrated that plasma DKK1 levels were significantly elevated in patients with acute COVID-19 and declined during clinical recovery (72). Experimental stimulation of human platelets with SARS-CoV-2 spike protein induced dose-dependent DKK1 release, an effect that was reduced by ACE2 inhibition, indicating that viral spike-ACE2 interactions may directly activate platelets to secrete DKK1 (72). Mechanistically, platelet-derived DKK1 appeared to amplify pulmonary inflammation by modulating adaptive immune responses. In SARS-CoV-2-infected hACE2 transgenic mice, elevated DKK1 was associated with altered T helper cell polarization, enhanced inflammatory cytokine production, and worsened lung pathology (72). Thus, platelet DKK1 inhibition during SARS-CoV-2 infections restores T-cell distribution, resulting in enhanced SARS-CoV-2 viral clearance and reduced lung damage (72). These findings support the concept that platelet activation during coronavirus infection extends beyond coagulation and thrombosis to include regulation of immune signaling pathways. The broader COVID-19 literature further supports a central role for platelet-driven thrombo-inflammation in disease severity (73, 74). SARS-CoV-2 induces platelet activation, platelet-leukocyte aggregation, P-selectin expression, and prothrombotic responses that contribute to endothelial injury and immunopathology (75).

In another study, it was demonstrated that platelet-derived DKK1 is an essential regulatory molecule in pulmonary T-cell immune responses during pulmonary infections, promoting Th2 and Th17 cell differentiation (76). Given the established ability of DKK1 to regulate Wnt signaling and inflammatory cell differentiation, platelet-derived DKK1 may function as an important intermediary linking viral sensing, platelet activation, immune dysregulation, and tissue injury during coronavirus infection. Collectively, these observations identify platelet-derived DKK1 as a potential biomarker and therapeutic target in COVID-19 and possibly other viral infections characterized by excessive immune-mediated tissue damage.

## Platelet-derived DKK1 and fungal infection

Previous studies explore the immunological role of platelets beyond hemostasis, identifying platelet-derived DKK1 as an important mediator of antifungal immunity during *Candida albicans* infection (76). In a murine model of airway mycosis, it was revealed that *Candida albicans* induces platelet activation through candidalysin, a fungal peptide toxin secreted during hyphal formation (76). Candidalysin engaged the platelet receptor GP1bα, triggering the release of platelet-derived DKK1 (76). Platelet-derived DKK1 promoted polarization of both Th2 and Th17 immune responses, which were associated with enhanced fungal clearance and reduced pulmonary fungal burden (76). The study further showed that depletion of platelets or interruption of the GP1bα-DKK1 axis impaired adaptive immune activation, leading to uncontrolled fungal invasion and severe pulmonary hemorrhage (76). These findings suggest that platelet DKK1 functions as a bridge between innate platelet sensing of fungal pathogens and downstream adaptive immunity. This work challenges the traditional perception of allergic Th2 responses as purely pathological. In the context of *Candida albicans* airway infection, platelet-mediated Th2/Th17 responses appeared protective, limiting fungal dissemination while preserving tissue integrity. Thus, platelet-derived DKK1 may represent a context-dependent immunomodulator capable of shaping host defense during fungal infection.

## How the release of platelet-DKK1 is regulated

Platelets possess three distinct types of granules (alpha (α), dense (δ), and lysosomes), each of which stores specific bioactive molecules (46). Platelet α-granules, the most abundant type of secretory granule, are now understood to be highly heterogeneous rather than uniform storage organelles, displaying substantial variation in their cargo content, structural characteristics, and functional roles. Although they were once thought to be homogeneous, advances in imaging techniques have demonstrated the presence of distinct α-granule subsets, enabling selective and differential release of their contents (77–79). This functional and structural diversity potentially enables platelets to act as dynamic sensors, releasing tailored sets of proteins in response to immediate needs, such as during various stages of clot formation, inflammation, or angiogenesis (80, 81). Numerous distinct membrane-bound or soluble proteins (such as DKK1) have been identified in α- granules, and are released into the extracellular space or expressed on the platelet surface upon activation (82). Dysregulated secretion of platelet granule contents plays a central role in mediating physiological and pathological responses associated with thrombotic, inflammatory, and infectious diseases (82). Various pathogens (53) are known to interact directly with various platelet surface molecules/receptors (TLRs, CLEC-2, DC-SIGN, GP-1b), which can lead to activation and granule exocytosis. Exocytosis of α-granule content is initiated and regulated by the activation of cell surface receptors (83). In the context of candidiasis, a previous study showed that candidalysin acts on platelets via the von Willebrand factor receptor GP1balpha (GP1bα) to stimulate the release of DKK1, which coordinates the development of Th2 and Th17 cell responses during airway mycosis caused by *Candida albicans* (76). In addition, *in vitro* studies demonstrated that the ancestral SARS-CoV-2 spike (S) protein induces DKK1 release from human platelets via the angiotensin-converting enzyme 2 receptor (ACE-2) (72). Evidence from the BALB/c *L. major* model confirmed that platelet DKK1 production primarily depends on the interaction between TLR1/2 and the *Leishmania*-derived LPG (84). Further, the stimulation of human platelets with soluble *Leishmania* antigen induces DKK1 production within 1 hour in a dose-dependent manner (54). While TLR4 is a major receptor for platelet activation (85), evidence of direct TLR4-induced DKK1 expression is not established (84). Although these studies established that the initiation of DKK1 secretion is regulated by activation of platelet surface receptors (72, 76, 84), the various proteins and processes that modulate DKK1 release from α-granules are poorly understood. In platelets, α-granule secretion is triggered by intracellular calcium (86). This process involves rapid exocytosis, in which granules dock, fuse with the plasma membrane, and release their contents. The regulation of α-granule exocytosis is mediated by chaperone proteins, docking factors, actin cytoskeleton, and soluble N-ethylmaleimide-sensitive factor attachment protein receptor proteins (80). Thus, it is likely that DKK1 secretion following activation of platelet surface receptors occurs through the fusion of α-granules with the plasma membrane. However, the precise ligands, receptors, and intracellular signaling pathways that regulate the release of platelet-derived DKK1 from α-granules remain poorly understood. In addition, the molecular mechanisms controlling platelet granule biogenesis, cargo sorting, and the selective packaging of proteins such as DKK1 into α-granules are still incompletely characterized.

## Immune response to leishmaniasis

Cutaneous leishmaniasis (CL), identified as a neglected tropical disease, is a vector-borne infection caused by protozoan parasites of the genus *Leishmania*. Despite sustained global efforts and collaborative initiatives over the past two decades, CL continues to account for 1–2 million new cases annually (87). The outcome of *Leishmania* infection hinges on the intricate equilibrium of pro- and anti-inflammatory host immune responses. The two arms of the immune system (innate and adaptive immunity) are closely linked, as cytokines released by innate immune cells shape the adaptive immune response to *Leishmania*. It is established that polarization of Th responses to either Th1 or Th2 can lead to life-or-death outcomes to infection with *L. major* in BALB/c mice (88, 89). Previous work has revealed that the antigen-presenting cell/co-stimulatory molecule/cytokine environment in which T-cells are primed influences Th polarization (88, 90, 91). Many of the cells that promote early parasite proliferation (neutrophils) and deliver activation signals to T cells (antigen-presenting cells such as dendritic cells and macrophages) are members of the innate immune system. (90, 91). However, there is limited knowledge on the upstream events that drive innate and adaptive immune responses during leishmaniasis. Platelets are now recognized as essential players in infection, where they disrupt tissue integrity and contribute to inflammation (92).

## Platelet-DKK1 influences innate immune cell response to *Leishmania* infection

Although platelets play a critical role in hemostasis, they are also rapidly recruited to infection sites, where they modulate immune responses by releasing cytokines, chemokines, and other inflammatory mediators (53, 93, 94). By interacting with other immune cells, platelets serve as important executors during inflammatory responses (95). For instance, activated platelets are known to express glycoprotein CD62P (a member of the selectin family) (96) following its release from the secretory α-granules. P-selectin glycoprotein ligand-1 (PSGL-1), a well-characterized selectin ligand, is primarily expressed in leukocytes (97). Leukocyte platelet aggregation (LPA), formed by adhesion between mature leukocytes and activated platelets, is mediated by PSGL-1 and CD62P, respectively (98, 99). LPA formation is required for the infiltration of cells to the inflammatory site (100). In the context of *Leishmania* infection, it has been demonstrated that the surface LPG (lipophosphoglycan) component of *L*. *major* activates platelets via TLR1/2, inducing P-selectin expression and DKK1 release from α- granules (84). In addition, available evidence indicate that platelet-derived DKK1 released in response to *L. major*-derived LPG is important in determining the course of leishmaniasis by modulating the recruitment and interactions of innate immune cells at the inflammatory site (101).

## Role of platelet-DKK1 on neutrophilic response to *Leishmania*

**Figure 1:**
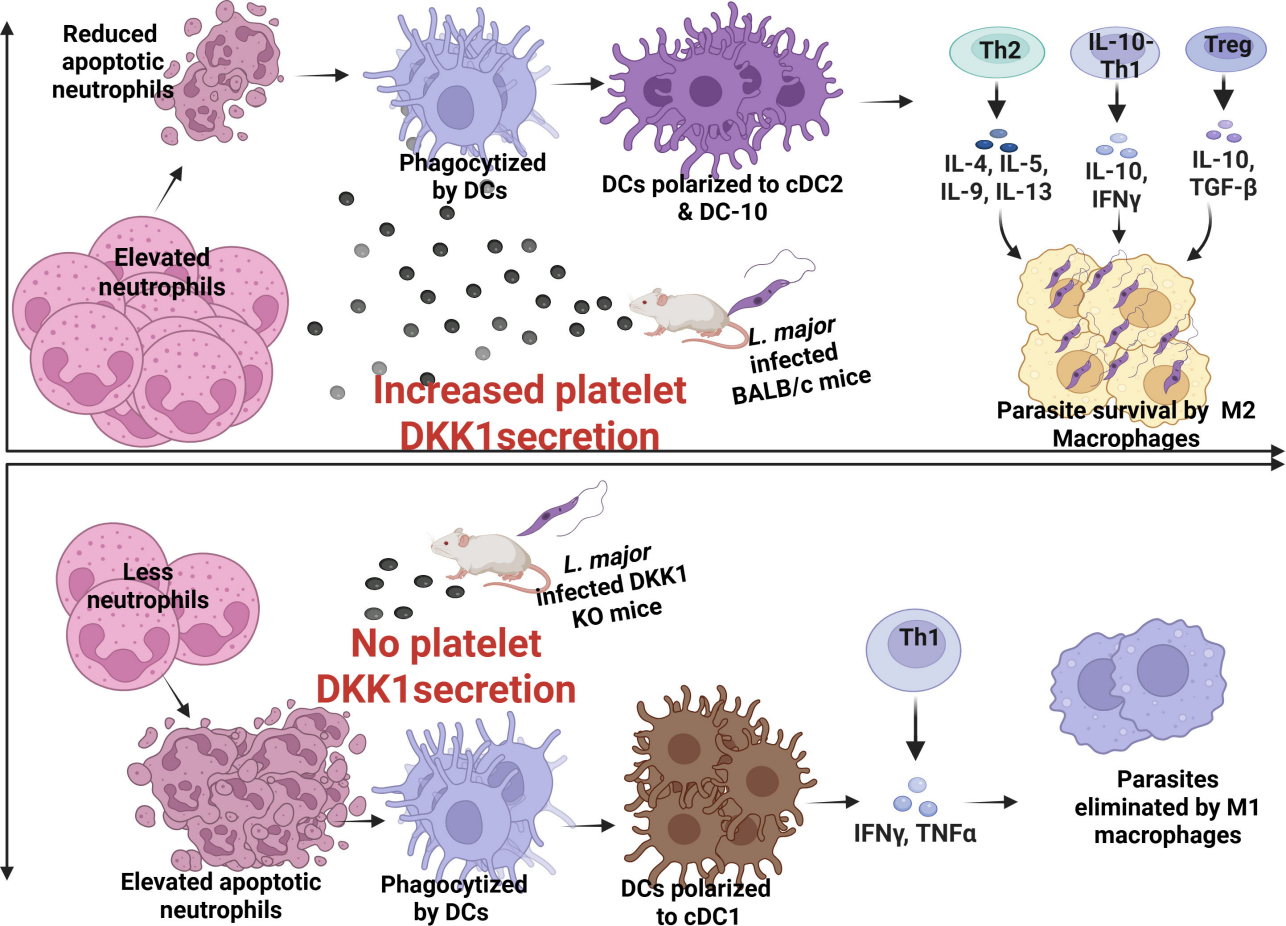
Proposed model based primarily on experimental BALB/c *L. major* model showing that *Leishmania* infection modulated innate and adaptive immune response to promote parasite survival in M2 macrophages of BALB/c infected mice. However, parasite elimination by M1 macrophages is seen in mice deficient in DKK1. Created in BioRender. Ihedioha, O. (111) https://BioRender.com/6utde7i.

Polymorphonuclear leukocytes are abundant in the circulation and have been regarded as the first line of defense in the innate arm of the immune system (102, 103). They continuously patrol the organism for signs of microbial infection, and when detected, these cells rapidly respond by capturing and destroying the invading microorganisms. Although inflammatory macrophages can actively phagocytize promastigotes injected by the insect vector, such primary encounters are less likely, as polymorphonuclear leukocytes, which can also capture promastigotes, are the first inflammatory cells to invade the infection site and exert a major influence on the early trajectory of infection. While polymorphonuclear leukocytes are the earliest recruited inflammatory cells following sand fly inoculation, tissue-resident cells such as dermal macrophages, dendritic cells, Langerhans cells, mast cells, and stromal cells are already present at the infection site and may interact with *Leishmania* promastigotes immediately after inoculation. Intravital microscopy studies demonstrated that polymorphonuclear leukocytes appear less than 1 minute following host exposure to sand flies (104). Additionally, activated polymorphonuclear leukocytes rapidly infiltrate the infection site following needle inoculation of parasites (105, 106). A variety of factors derived from the parasite influence the migration of polymorphonuclear leukocytes and the onset of infection. For example, *Leishmania* promastigotes have been reported to actively facilitate the recruitment of polymorphonuclear leukocytes by inducing endothelial cells to produce granulocyte chemotactic factor and CXCL8 (an effective chemoattractant for polymorphonuclear leukocytes) (107, 108). In addition, host factors can actively induce the recruitment of polymorphonuclear leukocytes. Polymorphonuclear leukocytes exhibit a strong chemotactic response to CXC chemokines, including IL-8, which has been implicated in mediating the early recruitment of these cells to sites of parasite inoculation in humans (108, 109). Polymorphonuclear leukocyte recruitment may also be mediated by pro-inflammatory cytokines, including tumor necrosis factor (TNFα) and interleukin-17 (IL-17) (109, 110). Recent reports further established that platelet-derived DKK1, which is released in the first 72 hours of parasite infection, induced neutrophil-platelet aggregation (NPA), which is essential for early trafficking of polymorphonuclear leukocytes to the *L. major* infection site (**Figure 1**) (101). This observation is consistent with previous findings, which showed that DKK1 upregulates LPA and leukocytes in blood obtained from wild-type parasite-infected mice, and that pretreatment with a DKK1 inhibitor before infection with *L. major* reduced the elevation in LPA formation, subsequent polymorphonuclear leukocyte infiltration into the draining lymph node (54) and led to reduced parasite burdens. These findings provide direct evidence that platelet DKK1 contributes to disease progression by promoting early neutrophil migration to the infection site.

Naïve polymorphonuclear leukocytes are relatively short-lived cells, exhibiting a circulating half-life of approximately 6–19 hours, after which they undergo spontaneous apoptotic cell death (112, 113). It has also been established that *L. major* can modulate and extend the longevity of infected polymorphonuclear leukocytes by blocking spontaneous apoptosis (114–117). Although *Leishmania* parasites can delay apoptosis in multiple host cell types, the inhibition of neutrophil apoptosis is particularly significant, as neutrophils constitute an early component of the acute inflammatory response that can influence the subsequent outcome of infection. Studies using the BALB/c *L. major* model evaluated the hypothesis that the DKK1-LRP6 interaction activated during *Leishmania* infection can extend the life span of polymorphonuclear leukocytes; notably, increased apoptosis was observed in infected BALB/c mice with polymorphonuclear leukocytes genetically deficient in LRP6 (**Figure 1**). Thus, the increased longevity of polymorphonuclear leukocytes found in *Leishmania*-infected BALB/c mice suggests that DKK1-LRP6 signaling influences the viability of polymorphonuclear leukocytes (84). This effect was further confirmed *in vitro* using polymorphonuclear leukocytes treated with recombinant DKK1 (84). The non-apoptotic polymorphonuclear leukocytes isolated from infected BALB/c mice may comprise cells that phagocytosed *Leishmania* during the early hours post-infection, in which apoptosis was initially suppressed by DKK1 released from parasite-activated platelets. In support of the broader pro-inflammatory role of platelet-derived DKK1, previous study demonstrated that platelet-derived DKK1 promotes acute neutrophilic lung inflammation through the upregulation of endothelial adhesion molecules, particularly ICAM-1 and VCAM-1 (118). Using platelet-specific DKK1-deficient mice, the authors observed reduced neutrophil infiltration and attenuated pulmonary inflammation following inflammatory challenge, indicating that platelet-derived DKK1 mediates platelet-endothelial crosstalk during acute lung injury (118). These findings further suggest that DKK1 amplifies inflammatory signaling pathways that enhance leukocyte recruitment and vascular activation, thereby exacerbating tissue inflammation. It is important to note that much of the evidence supporting this DKK1-neutrophil axis currently derives from a relatively limited number of related experimental studies, largely performed in murine BALB/c models of cutaneous leishmaniasis. At present, independent replication and validation in other *Leishmania* species, host genetic backgrounds, or clinical settings remain limited. Likewise, whether similar platelet-DKK1-dependent mechanisms operate in human leishmaniasis has not yet been clearly established. Most available data originate from experimental mouse infections, and direct evidence linking platelet-derived DKK1 to neutrophil dynamics in human leishmaniasis is still lacking. Therefore, although current findings support a model in which platelet-derived DKK1 modulates early neutrophilic responses during *L. major* infection, additional studies are needed to determine the clinical relevance of this pathway in human disease and across diverse experimental systems.

## Role of platelet DKK1 on dendritic cell response to *Leishmania*

Dendritic cells are professional antigen-presenting cells that establish connections with T cells and drive the developing immune response. Dendritic cells’ interaction with pathogens is mediated by various pattern recognition receptors (PRRs), the most well-known of which are Toll-like receptors (TLRs) (119). By presenting pathogenic molecules via MHC molecules to the T-cells, dendritic cells provide signals that induce T-cell proliferation and activation (120). Dendritic cells are a heterogeneous population of cells that can be subdivided into two major categories: the plasmacytoid dendritic cells (pDCs), known for type I interferon synthesis, and the conventional dendritic cells (cDCs), characterized by antigen processing and presentation for T-cell priming (121). Two cDCs subsets (cDC1 and cDC2) are distinguished based on functional specialization and preferentially drive Th1 and Th2 responses, respectively (122). The production of IL-12 by cDC1 skews the differentiation of naïve T-cells toward host-protective Th1 cells (123). On the other hand, cDC2s prime naïve CD4+ T cells for Th2 or Th17 polarization (124, 125). Several studies have reported a central role for dendritic cells in orchestrating the immune response against *Leishmania* infection (126, 127). Antigen presentation and IL-12 production by cDC1 are required for the differentiation of CD4^+^ Th1 and CD8^+^ T cells, resulting in a protective immune response (128, 129), while cDC2 dendritic cells have been linked to the development of Th2 response and pathology in leishmaniasis (121, 130). Previous studies have shown that TIM-3 (T-cell immunoglobulin and mucin-domain containing-3) increases the abundance of cDC2s during infections such as *L. donovani* by promoting an immunosuppressive environment. This effect is mediated through enhanced interleukin-10 (IL-10) production, which leads to T-cell exhaustion and diminished immune responses (130). Studies investigating the platelet effects on dendritic cells have reported heterogeneous outcomes, depending on the progenitor cell and conditions. Co-incubation of active platelets with bone marrow or monocyte-derived dendritic cells promotes dendritic cell maturation and stimulates the secretion of interleukin IL-6 and IL-12 (131, 132). Platelets release mediators and molecules that directly influence dendritic cell maturation and activation. For instance, the release of soluble CD40L and interaction with dendritic cell CD40 promotes overexpression of costimulatory molecules on dendritic cells and increases cytokine production by dendritic cells; this process has been shown to result in *S. aureus* bacterial uptake and killing (133). However, other studies have reported that dendritic cells exposed to platelet-derived serotonin or activated platelets exhibit impaired differentiation, a marked decrease in their ability to stimulate T cells, and increased production of the immunoregulatory cytokine IL-10 (134, 135). In the context of *L. major*, it has been demonstrated that platelet DKK1 upregulates the infiltration of cDC2, DC-10 cells, and other leukocytes at the infection site in BALB/c mice (**Figure 1**) (111), thereby inducing Th2 responses. This suggests that platelet DKK1 regulates the immunosuppressive Th2 response to *Leishmania* infection by promoting the infiltration of cDC2 and DC-10 cells at the site of infection. However, further investigation is also needed to determine how platelet DKK1 upregulates cDC2 and DC-10 cells, as the underlying mechanisms remain incompletely understood. Additionally, independent validation in other mouse models is still limited, and the relevance of these findings to human cutaneous leishmaniasis has yet to be established. Nevertheless, modulation of cDC2 predominance through inhibition of platelet DKK1 production may represent a potential investigational immunomodulatory strategy for limiting Th2-biased immune responses and improving host resistance during *Leishmania* infection.

## Role of platelet DKK1 on macrophage response to *Leishmania*

Macrophages are innate immune cells characterized by a range of activation states (classically activated (M1) and alternatively activated (M2)) that impact disease progression (136). M1 macrophages are associated with pro-inflammatory responses, including production of cytokines such as interleukin IL-6, IL-1β, IL-12, and tumor necrosis factor-alpha (TNF-α). Alternatively, M2 macrophages are involved in anti-inflammatory responses, such as IL-10 and transforming growth factor-beta (TGF-β), which help resolve inflammation (137). In general, the M1 macrophage subtype can be dominant during the initial phases of infection, shifting to M2 dominance as the infection resolves (138).

The investigations of the biology of macrophage differentiation and polarization have primarily focused on soluble stimuli (such as cytokines and TLR ligands); however, the role of cellular interactions in macrophage reprogramming remains poorly explored. Platelet-derived factors play an important role in reprogramming macrophage phenotypes, maintaining a dynamic M1/M2 balance and promoting a timely M1-M2 shift (139). Although platelets promote macrophage polarization toward a pro-inflammatory phenotype and increase the survival of septic mice (140), platelet-conditioned medium was also shown to induce an anti-inflammatory phenotype in macrophages (141). Numerous studies have explored the *in vitro* response of murine macrophages to different specific platelet derivatives, and they support that platelet derivatives can promote anti-inflammatory activity and drive macrophage M2 polarization (142–146). Further, platelet-derived PF4 was demonstrated to control monocyte survival and differentiation (147) as well as the induction of a distinct subtype of macrophages termed M4 (148). Additionally, platelet-derived PGE_2_ can regulate monocyte inflammatory responses (149, 150). Consequently, platelets and their secretome can variably influence macrophage development and polarization (M1/M2). Data obtained from the BALB/c *L. major* experimental model demonstrated that platelet DKK1 promotes parasite replication and survival by modulating macrophage polarization toward an M2 phenotype (**Figure 1**) (111). In this regard, platelet DKK1 and other platelet-derived factors could play an important role in maintaining a dynamic M1/M2 balance and promoting a timely M1-M2 shift. Although this M1-M2 transition may contribute to the resolution of inflammation in certain contexts, in leishmaniasis it may instead facilitate parasite persistence and disease progression. It is not yet known whether platelet-derived DKK1 directly influences macrophages or acts indirectly through other platelet-derived mediators and inflammatory pathways. This represents a significant knowledge gap that has not yet been adequately addressed. Although current evidence is primarily derived from the BALB/c *L. major* model, which supports an association between platelet-derived DKK1 and M2-like macrophage polarization, independent validation across other *Leishmania* species, diverse host genetic backgrounds, and human disease settings remains limited and warrants further investigation. Thus, research on the interactions of macrophages and platelet derivatives could provide relevant information on the function and mechanisms of the immune response.

Beyond the context of leishmaniasis, adipose-derived stem cells (ADSCs) have been shown to produce DKK-1 which enhances cutaneous wound healing by promoting M2 macrophage polarization (151). This effect is mediated through activation of the PI3K/AKT and JNK signaling pathways, underscoring a broader immunomodulatory role associated with DKK1-related signaling in tissue regeneration (151). Similarly, a recent study demonstrated that the anti-DKK1 monoclonal antibody DKN-01 suppresses gastric cancer progression by activating the cGAS-STING pathway, thereby enhancing antitumor immune responses (152). Notably, DKN-01 also reverses tumor-associated macrophage polarization by inhibiting M2-like immunosuppressive phenotypes, ultimately reshaping the tumor microenvironment toward a more pro-inflammatory and anti-tumor state (152). Together, these findings highlight DKK1 as a context-dependent immunomodulatory factor that can either promote tissue repair through M2 macrophage polarization or contribute to immunosuppressive microenvironments in cancer, making it a potentially important but double-edged therapeutic target across inflammatory, regenerative, and tumor settings.

## DKK1 modulates T-cell response to *Leishmania* infection

The abundance of pro- and anti-inflammatory cytokines characterizes the immune response during *Leishmania* infection. These cytokine profiles generated by T-cells are associated with the healing process (153–155) or disease development during *L. major* infection (156–158). The predominance of the Th1 response is observed in resistant mice infected with the parasite (159). Th1 cytokines activate leishmanicidal mechanisms in infected macrophages, with high levels of nitric oxide and reactive oxygen species, leading to parasite killing (160). However, susceptible BALB/c mice infected with *Leishmania* develop non-healing, progressive lesions characterized by a predominance of a Th2 immune response. Both CD4+ and CD8+ T cells contribute to immunity to leishmaniasis, and both can produce the essential effector cytokine IFN-γ (161–163). While CD4 T cells are established as the primary producers of IFN-γ upon *in vitro* restimulation and can promote parasite killing by activated macrophages (153), induction of the Th1 response partly depends on the activation of IFN-γ- producing CD8^+^ T cells (164, 165).

Upon activation, platelets demonstrate notable immunomodulatory capacity, as indicated by their ability to promote lymphocyte proliferation, modulate the lymphocyte subset balance through regulation of CD3^+^CD8^+^ T cell populations, and direct the differentiation of T-helper and Tregs (regulatory T) cells (166–169). Some of the immunoregulatory factors released by platelets exhibit immunosuppressive properties. Platelets and their soluble mediators can impede T-cell priming and differentiation into the IFNγ+ Th1 phenotype by monocyte-derived dendritic cells (170). For instance, platelets are the primary source of TGF-β, and this cytokine has been shown to have deleterious effects on various lymphocyte populations. Specifically, TGF-β inhibits T-cell differentiation into cytotoxic T cells while increasing the population of Tregs (blocking effector T cells and NK cell activity). TGF-β also directly affects NK cells by impairing their lytic activity and reducing IFN-γ production (171). Platelet-derived PF4 has been shown to inhibit nonregulatory T‐cell proliferation (172), and also to enhance regulatory T (Treg) cell differentiation and interleukin‐10 (IL‐10) secretion (173, 174). Recent evidence revealed that platelet DKK1 preferentially induces Th2 cytokines (IL-10), whereas the absence of platelet DKK1 promotes an IFN-γ response during *Leishmania* infection in the susceptible BALB/c mouse model (54, 111). Using direct intracellular cytokine staining, the influence of platelet DKK1 on the relative contribution of T-cell subsets in producing these cytokines (IFN-γ and IL-10) were further evaluated. A higher percentage of CD4^+^ and CD8^+^ T-cell-producing IFN-γ was observed in platelet DKK1-deficient infected BALB/c mice. However, there were higher levels of IL-10-producing CD4^+^ and CD8^+^ T-cells, as well as a low percentage of IFN-γ-producing CD4^+^ and CD8^+^ T-cells in the infected BALB/mice. In addition, the percentage of IL-10-Th1 cells was elevated in BALB/c-infected mice compared to platelet DKK1-deficient infected BALB/c mice (**Figure 1**). This suggests that platelet-derived DKK1 promotes disease progression by inducing the polarization of immune-suppressive T-cells in BALB/c-infected mice.

Interleukin 10 (IL-10), produced by a variety of cells (macrophages, regulatory T cells, Th1 cells, and CD8+ T-cells) during *L. major* infection, downregulates pro-inflammatory responses, primarily those induced by IFN-γ. An abundance of natural and inducible regulatory T cells in the lesions of patients with cutaneous leishmaniasis, as well as IL-10 and TGF-β production, has been observed (175–177). These cytokines contribute to disease pathology by deactivating macrophage killing mechanisms, leading to parasite persistence. Earlier studies showed that platelet DKK1 exhibits two distinct biological functions in the development of Foxp3+ Tregs. First, platelet DKK1 induced Foxp3+ Treg proliferation without inhibiting its suppressor function (54). Second, platelet DKK1 induced IL-10 expression by further inducing c-Maf in Foxp3+ Treg cells (54). It has been demonstrated that Treg cells in *L. major*-infected mice suppress effector T cell-mediated immunity and accumulate in the lesion site (178, 179). Furthermore, anti-CD25 treatment depletes Treg cells in the BALB/c mouse model of *L. major*, resulting in lesion size reduction and decreased disease severity (178, 180).

These findings collectively suggest that platelet-derived DKK1 may shift the immune response away from protective IFN-γ-dominated effector immunity and toward IL-10- regulatory or immunosuppressive phenotypes. Although the detection of IL-10, TGF-β, and regulatory T cells in *L. major* infected human supports the relevance of immunosuppressive pathways, these findings do not establish platelet-derived DKK1 as an upstream regulator in human leishmaniasis. Consequently, platelet DKK1 and other platelet derivatives may promote immunosuppressive T cells, thereby compromising effective anti-leishmania immunity.

## Conclusion

Neutrophils are among the first leukocyte populations recruited to sites of *Leishmania* infection and may facilitate parasite persistence through the proposed “Trojan horse” mechanism (109, 181). At the same time, recent evidence indicates that DKK1 produced by activated platelets in response to LPG function as early inflammatory mediators capable of enhancing neutrophil recruitment to the site of *Leishmania* infection (101). In addition to LPG-induced platelet-derived DKK1, platelet activation following complement engagement can stimulate the release of platelet-derived growth factor (PDGF), which subsequently promotes the production of monocyte chemoattractant protein-1 (MCP-1/CCL2). This chemokine contributes to the recruitment of inflammatory monocytes to sites of *Leishmania* replication and inflammation (182). Notably, these inflammatory monocytes may accumulate very early after infection, potentially before substantial neutrophil infiltration occurs at the lesion site (182). Once recruited, these monocytes contribute to shaping the local inflammatory milieu and may further influence dendritic cell and T-cell polarization (182).

The conventional paradigm linking Th1 responses with protection and Th2 responses with susceptibility does not fully account for the complexity of experimental and clinical observations in leishmaniasis. In the *L. major* BALB/c mouse model, specific cellular IL-4Rα expression as well as IL-4 administration during later stages of infection has been associated with enhanced healing responses (183–185). Similarly, in human *L. mexicana* infection (chiclero ulcer), patients with chronic nonhealing lesions can still develop robust systemic Th1-type immune responses, indicating that the presence of a Th1 response alone may be insufficient for effective parasite clearance or lesion resolution (186). These observations suggest that disease outcome is determined by multiple interacting factors, including parasite species, host genetic background, the tissue microenvironment, the timing and magnitude of cytokine production, and the balance between local and systemic immune responses. Collectively, these findings demonstrate that protective versus pathological immune responses in leishmaniasis are highly context dependent and cannot be explained solely by a strict Th1/Th2 dichotomy. Although the proposed model suggests that M2-like macrophages display reduced leishmaniacidal capacity, thereby supporting parasite persistence and chronic infection, the direct mechanistic relationship between platelet-derived DKK1 signaling and ROS dysregulation in M2 macrophages has not yet been fully established experimentally in the proposed model.

In conclusion, platelet-derived DKK1 may contribute to the immunological environment that favors persistence of *L. major* by regulating leukocyte recruitment, dendritic cell polarization, macrophage activation states, and downstream T-cell responses. In this context, “control” of leishmaniasis can be mechanistically understood as the ability to redirect these immune pathways toward more protective anti-parasitic immunity while limiting immune conditions that promote parasite survival. While our understanding of the role of platelet DKK1 has advanced substantially, many knowledge gaps remain and represent areas for future research. For example, based on the signaling framework illustrated in the graphical abstract, platelet-derived DKK1 modulates dendritic cell programming, including cDC2 and regulatory DC-10-like phenotypes, thereby shaping downstream T-cell polarization. In this context, dendritic cell derived cytokines are expected to establish distinct immunological milieus that bias T-cell differentiation toward Th1, Th2, or Treg responses. However, within the platelet DKK1-DC-T cell axis described in the model of *Leishmania* infection, the precise cytokine profile directly attributable to DC subsets driving these divergent T-cell fates has not yet been defined and remains to be experimentally characterized.

In addition, many of the proposed DKK1-immune cell interaction axes have primarily been investigated in the BALB/c *L. major* experimental model, thus independent validation in other *Leishmania* species, host backgrounds, and human disease settings remains limited. Another major limitation is the context-dependent nature of DKK1 signaling. While suppression of DKK1 may enhance protective immunity in some settings, excessive inhibition could also amplify damaging inflammatory responses and immunopathology. In leishmaniasis, strong Th1 responses are necessary for parasite control, but uncontrolled inflammation can contribute to tissue destruction and lesion severity. Thus, therapeutic modulation of DKK1 would likely require precise tissue-specific targeting rather than complete systemic blockade. Further, DKK1 has pleiotropic biological functions beyond infection, especially as a regulator of Wnt signaling, tissue homeostasis, bone metabolism, vascular biology, and immune regulation. Systemic inhibition of DKK1 could therefore produce unintended effects, including altered tissue repair, impaired wound healing, dysregulated inflammation, or skeletal complications. The heterogeneity of *Leishmania* species, host immune backgrounds, and disease manifestations further complicates the translation of DKK1-targeted approaches into broadly effective therapies. These unresolved questions highlight important areas for future investigation into the regulation of platelet-derived DKK1 in infectious and inflammatory diseases.

Nevertheless, while DKK1 represents a promising immunomodulatory target, further mechanistic, translational, and clinical studies are necessary to determine whether therapeutic modulation can safely achieve immune control without inducing excessive immunopathology or systemic adverse effects. As the discovery of how DKK1 secretion influences the outcomes of leishmaniasis expands, this knowledge not only enhances the understanding of the immune-regulatory role of platelets but also offers fresh perspectives and approaches to treating associated infectious and non-infectious diseases. From a therapeutic perspective, targeting DKK1 could therefore represent a strategy to rebalance host immunity. Inhibition of platelet-derived DKK1 may reduce excessive Th2/Treg polarization, limit tolerogenic dendritic-cell activity, and promote more effective Th1-associated responses characterized by IFN-γ-driven macrophage activation. Such immune reprogramming could enhance nitric oxide-mediated parasite killing and improve parasite clearance. Therapeutically, DKK1 modulation might also complement existing antiparasitic drugs by altering the inflammatory microenvironment rather than directly targeting the parasite itself. This approach could be particularly relevant in chronic or non-healing forms of leishmaniasis where dysregulated immune responses contribute to disease persistence. The broader implication is that DKK1-directed immunomodulation may represent a host-directed therapeutic strategy applicable not only to leishmaniasis but also to other infectious diseases characterized by maladaptive inflammatory or immunoregulatory responses.
